# *Xochiquetzallia* (Asparagaceae, Brodiaeoideae), a new genus segregated from the paraphyletic *Dandya*

**DOI:** 10.3897/phytokeys.139.46890

**Published:** 2020-01-27

**Authors:** Jorge Gutiérrez, Teresa Terrazas

**Affiliations:** 1 Área de Biología, Departamento de Preparatoria Agrícola, Universidad Autónoma Chapingo, Carretera México-Texcoco km 38.5, Texcoco 56230, Estado de México, México Universidad Autónoma Chapingo Texcoco Mexico; 2 Instituto de Biología, Universidad Nacional Autónoma de México, Ciudad Universitaria, Coyoacán 04510, Ciudad de México, México Universidad Nacional Autónoma de México México Mexico

**Keywords:** Asparagales, geophyte, gynophore, Mexico, *Milla* clade

## Abstract

A new genus, *Xochiquetzallia*, for the Brodiaeoideae, Asparagaceae family is here proposed. A taxonomic analysis based on morphology highlights its synapomorphies. The characters that distinguish *Xochiquetzallia* are the absence of a pith in the gynophore and the presence of an entire stigma. The recognition of *Dandya purpusii* as a monotypic genus is supported by the development of a short floral tube (< 2 mm) and a pith in the gynophore, as well as a divided stigma shared with the other genera of the *Milla* clade, *Bessera*, *Jaimehintonia*, *Petronymphe* and *Milla*. A key to its taxonomic determination is given for both the *Xochiquetzallia* species and the *Milla* clade genera.

## Introduction

**Figure 1.**
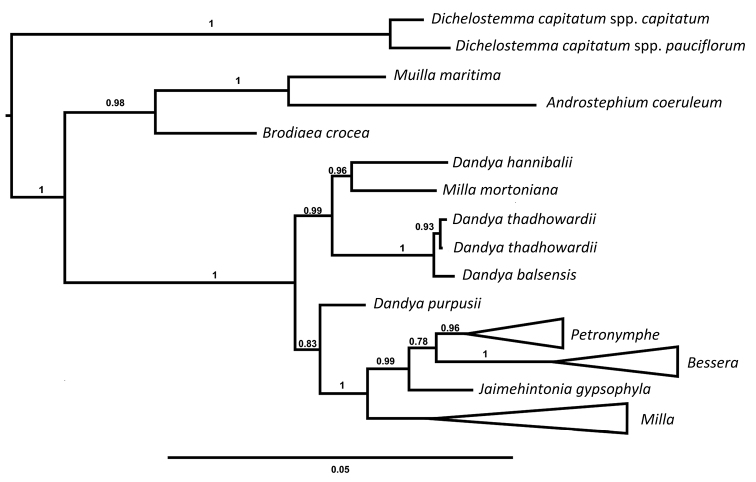
Phylogenetic relationships of the *Milla* complex genera (modified from Gutiérrez et al. 2017).

*Dandya* H.E. Moore is a genus endemic to Mexico and one of the five genera of the *Milla* clade (Gutiérrez et al. 2017). *Dandya purpusii* (Brandegee) H.E. Moore, the type species, has been placed in several genera in the past (Moore 1953) and molecular evidence suggest that generic limits in the complex are weak when few species are included in their phylogenetic analyses (Pires et al. 2001, Pires and Sytsma 2002, Gándara et al. 2014). Recently, Gutiérrez et al. (2017) conducted a study on the phylogeny of the *Milla* clade based on morphological, anatomical and molecular evidence (cDNA and ITS) and recovered two clades (Fig. 1). One clade recovers three species of *Dandya* plus *Milla mortoniana* strongly supported as sister to *Dandya purpusii* and the other four genera of the *Milla* clade (*Bessera*, *Jaimehintonia*, *Milla* and *Petronymphe*). Their results showed apomorphic structural characters that support the genera. The analyses clarify the phylogenetic relationships of genera and species of the *Milla* clade. The most relevant outcome is that *Dandya* is paraphyletic where three species of *Dandya* with distribution in the Balsas River Basin share the same ancestor as *Milla mortoniana* (Fig. 1). These four species (*D. balsensis*, *D. hannibalii*, *D. thadhowardii* and *M. mortoniana*) have as a synapomorphy the entire stigma. The genus *Milla* is paraphyletic due to the exclusion of *Milla mortoniana*. The members of *Milla* share four synapomorphies (thin pedicel, floral tubes > 60 mm, anthers with a bicollateral bundle and 20-30% of the ovary adnate to the floral tube) not present in *M. mortoniana*. *Dandya purpusii* shares with *Bessera*, *Jaimehintonia*, *Milla* and *Petronymphe* the dissected stigma, but no other character is mentioned. Based on Gutiérrez et al. (2017) results, here we identify characters that are consistent with the phylogeny and create a new genus for the clade of *Dandya* and a *Milla* species, *Dandya* is circumscribed, and taxonomic keys are given that allow differentiating the genera of the *Milla* clade and the new genus.

## Material and methods

Plant morphology was analyzed from field collected and herbarium material (ARIZ, BH, CHAPA, F, FC, GH, IEB, JEPS, MEXU, NY, RSA, UAMIZ, US and XAL). We studied the floral morphology from organisms collected in the field, except for *Milla mortoniana* (material removed from herbarium, MEXU). Morphological characters, vegetative and reproductive, were observed and analyzed with the help of a microscope (Nikon SMZ-2T) and terminology follows various authors (Moore 1953, Lenz 1971, Rahn 1998, Harris and Harris 2001). In addition, floral and foliar anatomical characters (Gutiérrez et al. 2010, 2015) were incorporated. The complete list of 60 characters and characters’ states used to delimit the monophyletic groups for the *Milla* complex can be consulted in Gutiérrez et al. (2017). Those characters that were recovered as synapomorphies or unique character combinations are here used to construct the keys for the *Milla* complex genera and the species of the new genus here proposed.

## Results

Based on the phylogenetic clades recovered by Gutiérrez et al. (2017) and the deep morphological analysis of the species, we proposed the amended circumscription of *Dandya* and the new genus *Xochiquetzallia*.

### Taxonomic treatment

#### Dandya

Taxon classificationPlantaeAsparagalesAsparagaceae

H.E. Moore (1951)

AFD28B63-9BE8-59FF-B005-D3B9721B031E

##### Diagnosis.

Perennial herbs, geophytic; terete; subcampanulate flowers, tube 1.5–3.0 mm long, erect; gynophore with pith, stigma divided.

#### Dandya purpusii

Taxon classificationPlantaeAsparagalesAsparagaceae

(Brandegee) H.E. Moore

AFAFD426-5411-5CA7-8FB5-E6EC0DD6409B

Dandya purpusii (Brandegee) H.E. Moore, Gent. Herb. 266–268 (1953).
Muilla purpusii Brandegee, Univ. Calif. Publ. Bot. iv, 177 (1911).
Bloomeria purpusii (Brandegee) Macbride, Contr. Gray Herb. Ser. 2. 56: 1-20 (1918).
Brodiaea purpusii (Brandegee) Ingram, Madroño xii, 27 (1953).

##### Description.

Perennial herbs, 28–47 cm tall, including corm and inflorescence. Fibrous roots. Corm subglobose compressed, fleshy, 1.0–1.7 cm in diameter; tunic formed by the wide bases of the leaves, brown or dark brown, covering up to 2.0 cm from the base of the scape. Leaves 2–3, 13–27 cm long, dark green, linear, subterete, with scabrous surface, hyaline prominences on the veins; base truncated, apex acute. Inflorescence in umbel; Scape of 27–42 cm long, usually longer than leaves, terete, surface smooth or with acute prominences. Floral bracts 2–3, linear-lanceolate, triangular, 4.0–8.5 mm long; bracteoles, one per flower. Flowers 6–11, pedicels 2.0–4.0 cm long, subcampanulate, erect, articulate, floral tube 1.5–3.0 mm long; tepals blue, 6 in 2 series, external tepals ovate-lanceolate, 6.0–10.0 × 1.5–3.0 mm, apex acute and papillose, base cuneate, entire margin; internal tepals ovate-lanceolate, 6.0–9.5 × 1.6–3.0 mm, apex obtuse and papillose, base cuneate, entire margin. Stamens 6; filaments free, adnate to the throat of the tube, widened toward the base, 4.0–6.0 mm; anthers oblongs, yellow, basifixed, 1.5–2.0 mm; gynophore 0.5–1.1 mm long, adnate to the floral perigone formed three cavities, present pith. Ovary cylindrical, 2.5–5.5 mm, fused at its base to the floral perigone; style filiform, 2.1–4.0 mm; stigma divided, papillose; capsule loculicidal, subcylindric, glabrous, brown, 10.0–12.0 mm long; seed oblong-falciform, compressed, black, bright, 3.0 × 5.0 mm.

##### Type species.

MEXICO. Coahuila; Sierra de la Paila, October 1910, *Purpus 4959* (holotype UC!; isotypes F!, GH!).

##### Specimen examined.

MEXICO: Coahuila, Municipality of Ramos Arispe, Valle de los Ángeles, Sierra de la Paila, 6 August 1975, *M. F. Robert* & *J. Passini 4675* (ENCB); El Cidral, Sierra de la Paila, 20 August 1987, *J. A. Villarreal 3980* (TEX); 5.9 miles east of the road between Yucatan and Mexico, highway 40, west of the mountains along a gravel road to 17.2 miles north of Mexico highway 40, 1650 m elevation, 19 September 1996, *J. M. Porter 11308* & *J. T. Columbus* (RSA); 15 km north of Estación Marte, on secondary road, 1550 m elevation, 24 October 2011, *J. Gutiérrez et al. 1225* (FEZA, CHAPA, MEXU, UAMIZ).

#### Xochiquetzallia

Taxon classificationPlantaeAsparagalesAsparagaceae

J.Gut. gen. nov.

E727D8F2-ABEF-5780-B5EA-B5E120EA55DA

urn:lsid:ipni.org:names:77204851-1

Fig. 2

##### Diagnosis.

Perennial herbs, geophytic; terete or flattened leaves; subcampanulate or hypocrateriform flowers, tube 1.0–25.0 mm long, erect or reclined; gynophore without pith, stigma entire.

##### Description.

Perennial herbs, 20–60 cm tall, including corm and inflorescence. Fibrous roots, some fleshy. Corm subglobose compressed, fleshy, 1.0–2.5 cm in diameter; tunic formed by the wide bases of the leaves, brown or dark brown, covering up to 2.0 cm from the base of the scape. Leaves 5–9, 20–49 cm long, dark green, linear, flattened or terete, with glabrous or scabrous surface, hyaline prominences on the veins; base truncated, apex acute. Inflorescence in umbel; Scape of (16–) 20–50 cm long, usually shorter than leaves, terete, surface smooth or with acute prominences. Floral bracts 2–3, linear-lanceolate, triangular, 3.0–9.0 mm long; bracteoles, one per flower. Flowers 4–20, pedicels 0.8–3.5 cm long, subcampanulate or hypocrateriform, erect or decumbent-descending, articulate, floral tube 1–25 mm long; tepals white or blue, 6 in 2 series, external tepals elliptic, 8.0–16.0 × (1.5–) 2.0–7.0 mm, 1–3 veins, apex acute and papillose, base cuneate, entire margin; internal tepals elliptical to broadly elliptical, (6.5–) 8.0–16.0 × (2.0–) 3.0–11.0 mm, apex obtuse and papillose, base cuneate, entire margin. Stamens 6; filaments free, adnate to the throat of the tube, widened toward the base or columnar, 2.0–5.0 (–7.0) mm; anthers linear, lanceolate-deltoid, yellow, basifixed, 1.0–2.5 mm; gynophore 0.8–1.6 mm long, adnate to the floral perigone formed three cavities. Ovary cylindrical, 1.0–5.0 mm, fused at its base to the floral perigone; style filiform, 1.8–7.0 mm; stigma entire, papillose; capsule loculicidal, subglobose or subcylindric, glabrous, brown, 6.0–13.0 mm long; seed oblong-falciform, compressed, black, bright, seed coat papillose, 4.0 × 1.5 mm.

**Figure 2.**
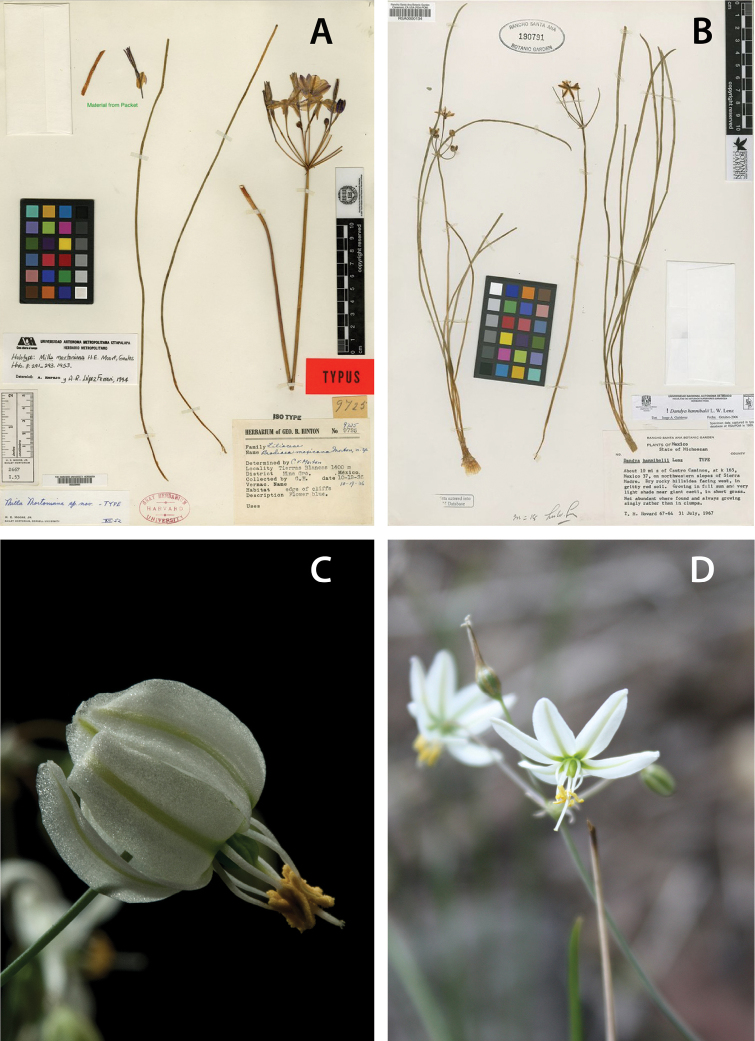
Species of the genus *Xochiquetzallia*. **A***Xochiquetzallia mortoniana***B***X. hannibalii***C***X. balsensis***D***X. thadhowardii*. **A** and **B** taken from the "Global Plants JSTOR".

##### Type species.

*Xochiquetzallia mortoniana* (H.E. Moore) J.Gut.

##### Etymology.

This genus is named in honor of the goddess of Aztec flowers, in Nahuatl "Xōchiquetzalli" (beautiful flower) "xṓchitl" (flower), "quétzalli" (beautiful). The Aztecs developed majestic architectural works, had extensive knowledge of astronomy and great respect for nature, particularly plants.

### Key to the taxonomic determination of the genera of the *Milla* clade

**Table:** 

1	Stigma entire	*** Xochiquetzallia ***
–	Stigma dissected	**2**
2	Connate stamens, dorsifixed anthers	*** Bessera ***
–	Free stamens, dorsifixed or subdorsifixed anthers	**3**
3	Subcampanulate flowers, gynophore < 2 mm	*** Dandya ***
–	Tubular flowers, gynophore > 2 mm	**4**
4	Flowers green-yellow, orange-red; tepals ascending or erect	*** Petronymphe ***
–	Flowers white, purple or pink; tepals divaricate	**5**
5	Diffuse-ascendant tepals; filaments 7-8 mm; floral tube < 15 mm long	*** Jaimehintonia ***
–	Divaricated tepals, occasionally reflexed; filaments < 7 mm; floral tube >15 mm long	*** Milla ***

### New combinations for *Xochiquetzallia*

#### Xochiquetzallia balsensis

Taxon classificationPlantaeAsparagalesAsparagaceae

(López-Ferr. & Espejo) J.Gut. comb. nov.

1F5640F9-71CB-5457-8953-FF6877A9F3C5

urn:lsid:ipni.org:names:77204852-1

**Figure 3.**
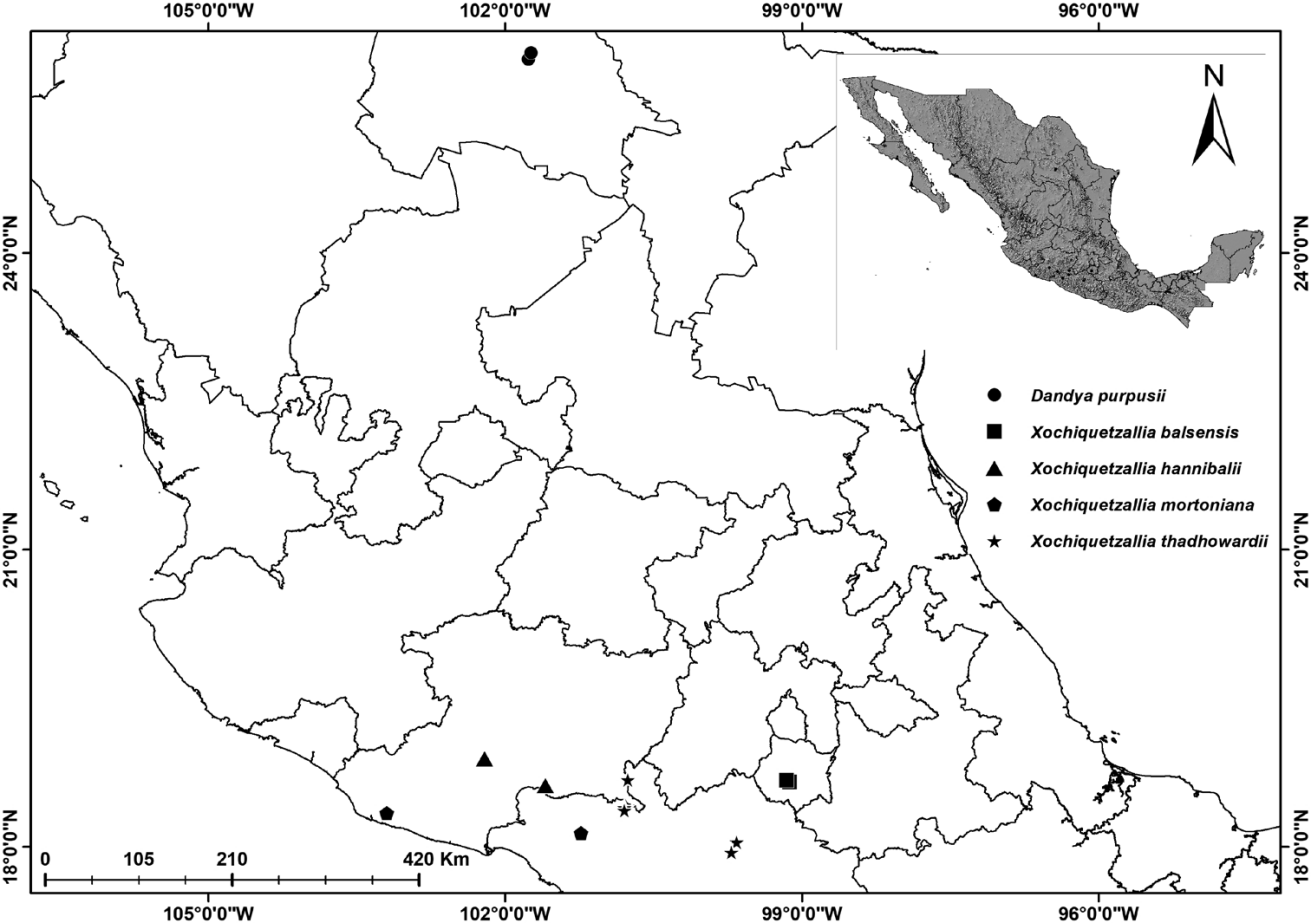
Geographical distribution of the species of *Xochiquetzallia* and *Dandya purpusii*.

≡ Dandya balsensis López-Ferr. & Espejo in Act. Bot. Mex. 18: 11–15, f. 1–2. (1992), basionym Type:– MEXICO. Morelos: Municipality Talquiltenango, road between Valle de Vázquez and Chimalacatlán, 1200 m elevation, 25 June 1989, *A. Flores Castorena 1075* & *D. Martínez Alvarado* (holotype, UAMIZ!) (Figs 2C, 3).

##### Specimens examined.

MEXICO, Morelos: Municipality Talquiltenango, road between Valle de Vázquez and Chimalacatlán, 1200 m elevation, 25 June 1989, *A. Flores Castorena 1075* & *D. Martínez Alvarado* (isotypes, ENCB! IEB!); Ibid, 06 July 2006, *J. Gutiérrez 797* (CHAPA, FEZA, UAMIZ, MEXU); 9 June 2007, *J. Gutiérrez 839* (CHAPA, FEZA, UAMIZ, MEXU).

#### Xochiquetzallia hannibalii

Taxon classificationPlantaeAsparagalesAsparagaceae

(L.W. Lenz) J.Gut. comb. nov.

D8016445-66BD-5388-8A38-78F301884BAC

urn:lsid:ipni.org:names:77204853-1

≡ Dandya hannibalii L.W. Lenz in Aliso 7(3): 316, f.2. (1971), basionym Type:– MEXICO, Michoacán: about 10 miles south of Cuatro Caminos, at km 165, Mexico 37, on the northwestern slopes of Sierra Madre, 31 July 1967, *T. M. Howard 67-64* (holotype, RSA 190791!) (Figs 2B, 3).

##### Specimens examined.

MEXICO, Michoacán: Municipality of Huetamo, Balsas road km 72, near Las Cruces bridge, 9 July 2006, *J. Gutiérrez 805* (CHAPA, FEZA, UAMIZ, MEXU); ibid., 2 September 2006, *J. Gutiérrez 813* (CHAPA, FEZA, UAMIZ, MEXU).

#### Xochiquetzallia mortoniana

Taxon classificationPlantaeAsparagalesAsparagaceae

(H.E. Moore) J.Gut. comb. nov.

E486FFDF-F030-5ECA-991C-01FB1FF64F23

urn:lsid:ipni.org:names:77204854-1

≡ Milla mortoniana H.E. Moore in Gentes Herbarum 8: 291 (1953), basionym Type: MEXICO, Guerrero: Distrito Mina, Tierras Blancas, 1400 m., 19 October 1936, *Hinton 9725* (holotype GH!; isotypes NY!, US!) (Figs 2A, 3).

##### Specimen examined.

MEXICO, Michoacán: Municipality of Aquila, *Sánchez-Mejorada et al. 4301* (MEXU!).

#### Xochiquetzallia thadhowardii

Taxon classificationPlantaeAsparagalesAsparagaceae

(L.W. Lenz) J.Gut. comb. nov

F494C13E-848B-577A-AE25-1C361E6FEDE0

urn:lsid:ipni.org:names:77204855-1

≡ Dandya thadhowardii L.W. Lenz in Aliso 7(3): 314, f.1. (1971), basionym Type: MEXICO, Guerrero: About 25-30 miles south of Iguala, on hillsides in calcareous soil, at km 216 on Mexico 95, July 1964, 1965, 1966, *Howard 64-74* (holotype!, RSA 100784) (Figs 2D, 3).

##### Specimens examined.

MEXICO, Guerrero: Municipality of Cutzamala de Pinzón, 3 km north of Cutzamala river, road to Bejucos, 340 m elevation, 21 July 1986, *A. Espejo 2481* & *T. Chehaibar* (UAMIZ); Municipality of Eduardo Neri, Barranca de Xococoapa, 1000 m elevation, 20 July 1991, *S. Peralta et al. 231* (FCME); Municipality of Xochipala, Llano Delgado, 1035 m elevation, 21 July 1991, *M. Gual 260* (FCME); km 62 highway Iguala-Chilpancingo, 910 m elevation, 4 July 1980, *Campos & Castelo 56* (FCME); ibid., *Campos & G. Velázquez 118* (FCME); 6 km east-northeast of Xochipala, 2 July 1980, *J. Saldivar & Sánchez s. n.* (FCME); ibid., *Velázquez Toledo & Campos 63* (FCME); ibid. on the northwest hillside, 13 July 1991, *M. Luna Flores 43* (FCME); Km 169 highway Iguala-Chilpancingo, 9 June 2007, *J. Gutiérrez 840* (CHAPA, FEZA, UAMIZ, MEXU); Highway Iguala-Chilpancingo, 2 km towards Filo de Caballo, 9 June 2007, *J. Gutiérrez 841* (CHAPA, FEZA, UAMIZ, MEXU); Municipality of Coyuca de Catalán, 3 km west of Coyuca de Catalán, 10 June 2007, *J. Gutiérrez 844* (CHAPA, FEZA, UAMIZ, MEXU). Michoacán: Municipality of Huetamo de Nuñez, 500 m towards Petachícuaro, 430 m elevation, 20 July1986, *A. Espejo 2467 T. Chehaibar* (UAMIZ); Petachícuaro, 9 km north of Huetamo, 400 m elevation, 9 July 1982, *José C. Soto* & *Esteban Martínez 4047* (ENCB, MEXU, UAMIZ); Municipality of San Lucas, highway Cd. Altamirano-San Lucas, km. 211, Rancho el Ovispo, 339 m elevation, 9 July 2006, *J. Gutiérrez 805* (CHAPA, FEZA, UAMIZ, MEXU); highway Cd. Altamirano-San Lucas, km. 188, 352 m elevation, 9 julio 2006, *J. Gutiérrez 806* (CHAPA, FEZA, UAMIZ, MEXU).

### Key to the taxonomic determination of the species of the genus *Xochiquetzallia*

**Table:** 

1	Flowers white, divaricate-recline	**2**
–	Flowers blue-violet, erect	**3**
2	Filaments ≤ 3 mm long; style ≤ 4 mm long; known from the State of Morelos	***X. balsensis***
–	Filaments > 3.0 mm long; style 4-6 mm long; known from the States of Guerrero and Michoacán	***X. thadhowardii***
3	Perianth tube ≤ 2 mm long; tepals ≤ 1 cm long; filaments ≤ 4 mm long	***X. hannibalii***
–	Perianth tube 2-2.5 cm long; tepals 1.5-1.6 cm long; filaments 2 mm long	***X. mortoniana***

### Note

*Xochiquetzallia balsensis* (*Dandya balsensis*) and *X. thadhowardii* (*D. thadhowardii*) present morphological similarity and have a sympatric geographic distribution (Fig. 3). López-Ferrari and Espejo-Serna (1992) pointed out that *Xochiquetzallia balsensis* differs from *X. thadhowardii* by the size of the perigonium, filament and style segments, smaller in *X. balsensis*. Also, these authors indicate that in *X. thadhowardii* the anthers are firmly united among them around the style whereas in *X. balsensis* they are free. No differences in the morphological and anatomical characters were found between both species (Gutiérrez 2009; Gutiérrez et al. 2010, 2015). The analysis carried out by Gutiérrez et al. (2017) shows that the population of the here proposed *Xochiquetzallia balsensis* from the state of Morelos is the only one that differentiates itself by the size of the filaments and the style as proposed by López-Ferrari and Espejo-Serna (1992). Based on our field observations in the type locality of *Xochiquetzallia balsensis* we confirm that their anthers are firmly united among them around the style as in *X. thadhowardii* and the separation of the anthers takes place in advanced stages of the anthesis, as occurs with *X. thadhowardii*. In this sense, it is concluded that *Xochiquetzallia balsensis* should be circumscribed by its filaments no larger than 3 mm long and less than or equal to 4 mm long.

## Discussion

In *Dandya* and *Xochiquetzallia*, the connate stamens have been a recurrent character in the descriptions (Moore 1953; Lenz 1971; López-Ferrari and Espejo-Serna 1992). The staminal connation has been described as "crown or cup" at the base of the stamens and it was considered a diagnostic character for *Dandya*. Gutiérrez et al. (2010) did not find connate stamens in *Dandya* and *Xochiquetzallia* in their floral development study of the *Milla* complex. Moreover, they discovered that what was called “crown or cup” is the base of the filaments that is wider than the upper part and gives the appearance of detaching when the stamens are released asynchronously. Gutiérrez et al. (2010), also showed that *Bessera* is the only genus of the *Milla* clade that has connate filaments.

The existence of gynophore has also been a controversial feature. A gynophore was not described for the former species of *Dandya* now in *Xochiquetzallia* (*X. balsensis*, *X. hannibalii* and *X. thadhowardii*; Lenz 1971; López-Ferrari and Espejo-Serna 1992). Gutiérrez et al. (2010), when studying the gynophore, checked if it exists in the *Xochiquetzallia* species. They found that, unlike other genera of the *Milla* clade, the gynophore is short (< 2 mm) and lacks a pith. Also, *Xochiquetzallia balsensis*, *X. hannibalii* and *X. thadhowardii* have an entire stigma, a character shared with *X. mortoniana* which also lacks a pith in the gynophore. A recent study of floral anatomy of *Dandya purpusii*, allowed us to confirm that this species does have a pith in the gynophore as also found in *Bessera*, *Jaimehintonia*, *Milla* and *Petronymphe* (Gutiérrez et al. 2017). The short floral tube (< 2 mm) of *Dandya purpusii* was previously used as evidence to classify this species with the species of Xochiquetzallia (Dandya) (*X. balsensis*, *X. hannibalii* and *X. thadhowardii*) (Lenz 1971; López-Ferrari and Espejo-Serna 1992). However, the presence of the pith and the dissected stigma were not considered, and now we know both characters present in *Dandya purpusii* are shared with the other four genera. *Dandya purpusii* is distinguished by its ephemeral flowering and has been scarcely collected after its description in 1911 (Brandegee 1911). Further explorations in the distribution area of *Dandya purpusii*, now recognized as the only species of the genus *Dandya*, will hopefully reveal more localities and potentially the discovery of other species that are difficult to locate due to their ephemeral reproductive biology. The disjunct distribution between *Dandya purpusii* and the species of *Xochiquetzallia* suggests that both genera evolved independently and converged on floral shape as an adaptation to pollinators, among them the Lepidoptera.

Moore (1953) considered the floral shape as a discrete character that allowed to separate the genera of the *Milla* complex; for example, diagnostic floral shapes include subcampanulate for *Dandya*, tubular in *Petronymphe*, tubular and campanulate in *Bessera*, and hypocrateriform in *Jaimehintonia* and *Milla*. *Xochiquetzallia mortoniana* presents hypocrateriform flowers and this character allowed Moore (1953) to classify this species as *Milla*. Our investigations here found that *Bessera* species have all floral shape variations described in the genera of the clade (*Bessera tuitensis* has subcampanulate flowers, *B. elegans* campanulate, and *B. tenuiflora* tubular). The floral shapes among *Xochiquetzallia* species are either hypocrateriform or subcampanulate.

## Supplementary Material

XML Treatment for Dandya

XML Treatment for Dandya purpusii

XML Treatment for Xochiquetzallia

XML Treatment for Xochiquetzallia balsensis

XML Treatment for Xochiquetzallia hannibalii

XML Treatment for Xochiquetzallia mortoniana

XML Treatment for Xochiquetzallia thadhowardii
